# The synthetic oleanane triterpenoid CDDO‐2P‐Im binds GRP78/BiP to induce unfolded protein response‐mediated apoptosis in myeloma

**DOI:** 10.1002/1878-0261.13447

**Published:** 2023-06-13

**Authors:** George Luo, Kristin Aldridge, Toby Chen, Vivek Aslot, Byung‐Gyu Kim, Eun Hyang Han, Neelima Singh, Sai Li, Tsan Sam Xiao, Michael B. Sporn, John J. Letterio

**Affiliations:** ^1^ Department of Pathology Case Western Reserve University School of Medicine Cleveland OH USA; ^2^ Triterpenoid Therapeutics Inc. Tunbridge VT USA; ^3^ Trinity College of Arts and Sciences Duke University Durham NC USA; ^4^ Department of Chemical and Biomolecular Engineering Case Western Reserve University Cleveland OH USA; ^5^ The Angie Fowler Adolescent and Young Adult Cancer Institute University Hospitals Rainbow Babies & Children's Hospital Cleveland OH USA; ^6^ The Case Comprehensive Cancer Center Cleveland OH USA; ^7^ Department of Pediatrics Case Western Reserve University Cleveland OH USA

**Keywords:** apoptosis, CDDO‐2P‐Im, GRP78, myeloma, triterpenoid, UPR

## Abstract

Synthetic oleanane triterpenoids (SOTs) are small molecules with broad anticancer properties. A recently developed SOT, 1‐[2‐cyano‐3,12‐dioxooleana‐1,9(11)‐dien‐28‐oyl]‐4(‐pyridin‐2‐yl)‐1H‐imidazole (CDDO‐2P‐Im or ‘2P‐Im’), exhibits enhanced activity and improved pharmacokinetics over CDDO‐Im, a previous generation SOT. However, the mechanisms leading to these properties are not defined. Here, we show the synergy of 2P‐Im and the proteasome inhibitor ixazomib in human multiple myeloma (MM) cells and 2P‐Im activity in a murine model of plasmacytoma. RNA sequencing and quantitative reverse transcription PCR revealed the upregulation of the unfolded protein response (UPR) in MM cells upon 2P‐lm treatment, implicating the activation of the UPR as a key step in 2P‐Im‐induced apoptosis. Supporting this hypothesis, the deletion of genes encoding either protein kinase R‐like endoplasmic reticulum kinase (*PERK*) or DNA damage‐inducible transcript 3 protein (*DDIT3*; also known as *CHOP*) impaired the MM response to 2P‐Im, as did treatment with ISRIB, integrated stress response inhibitor, which inhibits UPR signaling downstream of PERK. Finally, both drug affinity responsive target stability and thermal shift assays demonstrated direct binding of 2P‐Im to endoplasmic reticulum chaperone BiP (GRP78/BiP), a stress‐inducible key signaling molecule of the UPR. These data reveal GRP78/BiP as a novel target of SOTs, and specifically of 2P‐Im, and suggest the potential broader utility of this class of small molecules as modulators of the UPR.

## Graphical abstract

The synthetic oleanane triterpenoid CDDO‐2P‐Im selectively interacts with specific protein targets in a concentration‐dependent manner. At low concentrations, its binding to KEAP1 leads to the activation of Nrf2 target gene expression. As the concentration of CDDO‐2P‐Im increases, it binds to GRP78, leading to the activation of the unfolded protein response and the upregulation of ATF4 and XBP1 target genes.

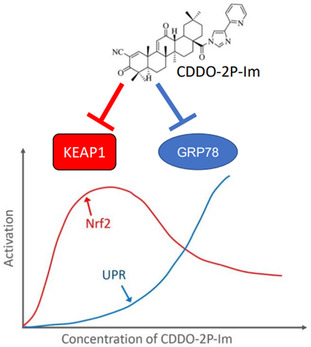

**Abbreviations**
ATF4: activating transcription factor 4
ATF6: activating transcription factor 6
BiP: binding immunoglobulin protein
CDDO: 1‐[2‐cyano‐3,12‐dioxooleana‐1,9(11)‐dien‐28‐oyl]
CDDO‐2P‐Im: 1‐[2‐cyano‐3,12‐dioxooleana‐1,9(11)‐dien‐28‐oyl]‐4(‐pyridin‐2‐yl)‐1H‐imidazole
CHOP: C/EBP homologous protein
c‐MYC: c‐myelocytomatosis oncogene product
DARTS: drug affinity responsive target stability
eIF2a: eukaryotic translation initiation factor 2A
ER: endoplasmic reticulum
GRP78: glucose‐regulated protein 78
IL‐6: interleukin 6
IRE1α: inositol‐requiring enzyme 1 α
ISRIB: integrated stress response inhibitor
KEAP1: Kelch‐like ECH‐associated protein 1
MM: multiple myeloma
mTOR: mammalian target of rapamycin
Nrf2: nuclear factor E2‐related factor 2
PCA: principal component analysis
PERK: protein kinase R‐like endoplasmic reticulum kinase
PI: proteasome inhibitor
ROS: reactive oxygen species
SOT: synthetic oleanane triterpenoid
STAT: signal transducer and activator of transcription
UPR: unfolded protein response
XBP1: X‐box binding protein 1

## 1 Introduction

Oleanane triterpenoids represent a family of naturally occurring small molecules that are found in plants and that possess potent anti‐inflammatory and anti‐cancer properties [1, 2]. Synthetic Oleanane Triterpenoids (SOTs) such as 1‐[2‐cyano‐3,12‐dioxooleana‐1,9(11)‐dien‐28‐oyl]imidazole (CDDO‐Im) have been developed to increase the potency of these naturally occurring compounds while retaining the anti‐inflammatory and anti‐cancer activities, such as the capacity to inhibit proliferation and induce apoptosis in many types of cancers cells [3, 4, 5, 6]. Recently, a fourth‐generation SOT, 1‐[2‐Cyano‐3,12‐dioxooleana‐1,9(11)‐dien‐28‐oyl]‐4(‐pyridin‐2‐yl)‐1H‐imidazole (CDDO‐2P‐Im, or ‘2P‐Im’) has been developed [7], and characterized as having improved pharmacokinetics, bioavailability, stability and enhanced potency relative to the parent molecule, CDDO‐Imidazolide [8, 9, 10, 11, 12]. However, the exact mechanisms that mediate the effects of 2P‐Im and other SOTs have yet to be defined.

We previously demonstrated the capacity of CDDO‐Im to induce apoptosis in human multiple myeloma (MM) cells [13], through mechanisms that included suppression either of the constitutive or of the interleukin 6 (IL‐6) induced phosphorylation of signal transducers and activators of transcription (STATs), specifically STAT3 and STAT5. These effects were linked to the upregulation of critical inhibitors of STATs, such as suppressors of cytokine signaling‐1 and SH2‐containing phosphatase‐1 (a tyrosine phosphatase). However, subsequent proteomic studies have shown the mammalian target of rapamycin (mTOR) is also a direct target of CDDO‐Im [10], and that binding to mTOR inhibits its kinase activity. These studies have been corroborated by other laboratories which have demonstrated induction of apoptosis by CDDO‐Im occurs in either the presence or absence of bone marrow stromal cells [14], and that CDDO‐Im acts synergistically with the proteasome inhibitor (PI) bortezomib to induce apoptosis in human myeloma cells, including in populations that are bortezomib‐resistant [14]. The importance of these observations is supported by studies that have shown the induction of Activating Transcription Factor 4 (ATF4) and of the C/EBP Homologous Protein (CHOP) in MM requires mTORC1 regulation of c‐myelocytomatosis oncogene product (c‐MYC), and that this signaling pathway is a major determinant in the ability of bortezomib to induce apoptosis [15].

In MM, the induction of ATF4/CHOP reflects an activation of the unfolded protein response (UPR), a signaling pathway activated by the accumulation of misfolded proteins within the endoplasmic reticulum (ER) [16]. The UPR is composed of three branches which have the following signaling proteins: Activating Transcription Factor 6 (ATF6), protein kinase R (PKR)‐like endoplasmic reticulum kinase (PERK), and inositol‐requiring enzyme 1 α (IRE1α) [17]. Each of these branches can be activated when the glucose‐regulated protein 78 (GRP78/BiP) is dissociated from the signaling proteins leading to the subsequent signaling cascade and transcriptional changes that constitute the UPR [18]. GRP78/BiP is an ER chaperone that is inherently expressed in MM, and collectively, these UPR components are required for B‐cell differentiation into antibody‐secreting cells [19, 20, 21]. Importantly, GRP78/BiP has been identified as a principal molecular target whose suppression by metformin effectively modulates autophagy to enhance the antimyeloma effects of bortezomib [22]. Taken together, these data define the rapid induction of components of the pro‐apoptotic/terminal UPR, such as ATF4, PERK, and the ER stress‐specific eIF‐2alpha kinase [23], as critical determinants of PI efficacy. Moreover, they highlight the potential utility of agents targeting GRP78/BiP to augment PI response and overcome PI resistance [23, 24, 25, 26].

Here we show GRP78/BiP is a direct target of 2P‐Im in MM cells. Although prior studies have shown the third generation SOT, CDDO‐Methyl Ester (CDDO‐Me), can induce the UPR, and that inhibition of the UPR abrogates the induction of apoptosis by CDDO‐Me [27, 28], the data presented here provide the first demonstration of a specific mechanism through which an SOT activates the UPR. CDDO‐2P‐Im induced apoptosis of human MM at nanomolar concentrations, and acted synergistically with the PI ixazomib, a second‐generation oral PI for the treatment of MM while being more tolerable compared with first generation PI [29]. RNA‐Sequencing of CDDO‐2P‐Im treatment revealed activation of signaling pathways including reactive oxygen species (ROS), cell cycle arrest, and the UPR. However, quantitative reverse transcription PCR (qRT‐PCR) and protein expression analyses confirmed activation of the PERK and IRE1α branches of the UPR are associated with the induction of apoptosis. Furthermore, both drug affinity responsive target stability (DARTS) and thermal shift assay confirmed that GRP78 is a direct target of 2P‐Im, suggesting activation of the UPR is due to 2P‐Im binding to GRP78. Through gene deletion and pharmacologic inhibition of components of the PERK‐ATF4‐CHOP axis, we also define this branch of the UPR as linked to the induction of MM apoptosis by 2P‐Im. These observations suggest that both 2P‐Im specifically, and likely other SOTs, have the potential to be positioned as modulators of GRP78, and highlight the potential for their clinical applications beyond cancer [30].

## 2 Materials and methods

### 2.1 Cell culture

The myeloma cell line, RPMI‐8226 (RRID:CVCL_0014), and plasma cell leukemia line, ARH‐77 (RRID:CVCL_1072), were both purchased from American Type Culture Collection, ATCC (Manassas, VA, USA). The mouse myeloma, 5T33MM cells (RRID:CVCL_G080), were obtained from K. Vanderkerken [31, 32]. IXZ‐resistant RPMI‐8226 was generated from a previous study [33]. ARH‐77, RPMI‐8226, and 5T33MM cells were maintained in RPMI 1640 medium (Thermo Fisher Scientific, Waltham, MA, USA) supplemented with 10% heat‐inactivated fetal calf serum (GeminiBio, West Sacramento, CA, USA) and penicillin–streptomycin mixed solution (Thermo Fisher Scientific), at 37 °C in a 5% CO_2_ atmosphere. All cell lines including those genetically modified were authenticated using CWRU genomic core short tandem repeat validation (> 80% match) and tested for mycoplasma regularly.

### 2.2 Reagents and antibodies

CDDO‐2P‐Im (96.3% purity by HPLC) was generously donated by M. Sporn. Ixazomib was purchased from Selleckchem (Houston, TX, USA). Integrated stress response inhibitor (ISRIB) was purchased from Millipore Sigma (#509582; Burlington, MA, USA). Tunicamycin was purchased from Thermo Fisher (J62217.MA) DMSO and Cremophor EL was purchased from Thermo Fisher Scientific. Antibodies to p‐IRE1α (PA116927; Thermo Fisher Scientific), IRE1α (27528‐1‐AP; Proteintech, Rosemont, IL, USA), Caspase‐8 (13423‐1‐AP; Proteintech), β‐actin (66009‐1‐Ig; Proteintech), p‐PERK (PA5‐40294; Thermo Fisher Scientific), PERK (20582‐1‐AP; Proteintech), GRP78 (66574‐1‐Ig; Proteintech), ATF4 (10835‐1‐AP; Proteintech), ATF6 (66563‐1‐Ig; Proteintech) CHOP (15204‐1‐AP; Proteintech), GAPDH (60004‐1‐Ig; Proteintech), LONP1 (15440‐1‐AP; Proteintech) were purchased from reputable commercial vendors. Secondary immune‐fluorescent secondary antibodies to rabbit (926‐68073; Li‐cor, Lincoln, NE, USA) and mouse (926‐32212; Li‐cor) were also purchased from commercial vendors.

### 2.3 Determination of cell viability

Cells were incubated in 96‐well plates at a density of 2.5 × 10^4^ per well. Either the control (1% DMSO) or selected therapeutic agents were added to each well at the indicated concentrations, and cells were incubated for 24 h. CellTiter‐Glo™ (Promega Corp, Madison, WI, USA) was used to investigate cell viability according to the manufacturer's instructions. Cell viability was also evaluated by flow cytometry of Annexin V/propidium iodide staining. To do so, cells were seeded at 5 × 10^5^ in 6 well plates and subsequently incubated with either DMSO (1%) or the specified therapeutic agents for 24 h. Cells were stained with Annexin V (Catalog #640906; Biolegend, San Diego, CA, USA) and propidium iodide (Catalog #421301; Biolegend) after 24 h and analyzed according to the manufacturer's instruction. Experiments were done in triplicates.

Incucyte (Sartorius, Göttingen, Germany) was used to image cells. RPMI‐8226 cells were treated with DMSO or CDDO‐2P‐Im and then plated at 2.5 × 10^4^ per well in 96‐well plates and spun down using a centrifuge. Cell confluence was estimated using incucyte zoom software (Sartorius, Göttingen, Germany) and analyzed in Excel.

### 2.4 Computation of synergy and IC_50_

The data generated from assays of cell viability using CellTiter‐Glo™ was analyzed using compusyn, a computer program for quantitation of synergism and antagonism in drug combinations, and the determination of both IC50 and ED50 Values [34]. A combination index value of 1 means the two drug effects are additive whereas a value below 1 indicates the drugs are synergistic while values above 1 indicate drugs are antagonistic.

### 2.5 Mouse plasmacytoma models

All animal experiments were performed in accordance with guidelines and approval of Institutional Animal Care and Use Committees at Case Western Reserve University (Protocol #2019‐0034). C57BL/KaLwRij mice were purchased from Envigo (Indianapolis, IN, USA). 5T33MM cells were cultured and suspended in PBS at 1 × 10^7^ mL^−1^. Female and male mice of age 8–10 weeks were injected with 1 × 10^6^ cells in the right flank. Mice were treated with either vehicle (10% DMSO, 10% Cremophor EL, 80% PBS) or 24 mg·kg^−1^ CDDO‐2P‐Im solution (10% CDDO‐2P‐Im dissolved in DMSO, 10% Cremophor EL, 80% PBS) by oral gavage. Treatment occurred daily five times a week followed by 2 days of rest. Tumor volume was measured by a caliper and calculated with the formula ([width]^2^ × [length]/2). The body weight was also recorded throughout the experiment.

For immunoblotting experiments with heterotopic tumors, mice were injected with 1 × 10^6^ cells in the right flank. Tumors were allowed to grow for 14 days before treatment with either vehicle or 24 mg·kg^−1^ CDDO‐2P‐Im by oral gavage. Mice were sacrificed 12 h later for analyses of tumors. Tumors were lysed for protein using Cell Lysis solution (#9803; Cell Signaling, Danvers, MA, USA) and subsequently prepared for immunoblot.

Female and Male NSG (NOD scid gamma) mice of age 8–10 weeks were purchased from Case Western Reserve University Animal Core facility. Mice were injected with 1 × 10^6^ cells in the right flank. Mice were treated with either vehicle (10% DMSO, 10% Cremophor EL, 80% PBS) or 12 mg·kg^−1^ CDDO‐2P‐Im solution (10% CDDO‐2P‐Im dissolved in DMSO, 10% Cremophor EL, 80% PBS) by oral gavage. Treatment occurred daily for 3 days followed by 1 day of rest followed by three more treatments. Tumor volume was measured by a caliper and calculated with the formula ([width]^2^ × [length]/2). The body weight was also recorded throughout the experiment. Animals were handled in the Animal Resource Center facilities at Case Western Reserve University according to approved protocols. Mice were maintained in a 12–12 h light–dark cycle, kept under normal room temperature, and fed standard chow diet and water.

### 2.6 RNA‐sequencing and qRT‐PCR

Cells were incubated with either DMSO (1%) or the specified therapeutic agents for 6 h prior to RNA extraction using Purelink RNA kit (Thermo Fisher Scientific) followed by DNase I (Thermo Fisher Scientific) treatment. RNA was measured by Nanodrop (Thermo Fisher Scientific) and then either sent to a commercial sequencing service, LC Sciences, for RNA‐Sequencing or prepared for cDNA synthesis (#4368813; Thermo Fisher Scientific).

Poly(A) RNA‐sequencing library was prepared following Illumina's TruSeq‐stranded‐mRNA sample preparation protocol [35]. RNA integrity was checked with Agilent Technologies 2100 Bioanalyzer (Agilent, Santa Clara, CA, USA). Poly(A) tail‐containing mRNAs were purified using oligo‐(dT) magnetic beads with two rounds of purification. After purification, poly(A) RNA was fragmented using a divalent cation buffer at elevated temperature. Quality control analysis and quantification of the sequencing library were performed using Agilent Technologies 2100 Bioanalyzer High Sensitivity DNA Chip. Paired‐ended sequencing was performed on Illumina's NovaSeq 6000 (Illumina, San Diego, CA, USA) sequencing system.

cutadapt was used to remove the reads that contained contamination, low quality, and undetermined bases [36]. Then, sequence quality was verified with fastqc (http://www.bioinformatics.babraham.ac.uk/projects/fastqc/). hisat2 was used to map reads to the genome of ftp://ftp.ensembl.org/pub/release‐96/fasta/homo_sapiens/dna/ [37]. The mapped reads were assembled using stringtie [38]. The transcriptomes were merged to reconstruct a comprehensive transcriptome using perl scripts and gffcompare. After the final transcriptome was generated, stringtie and edger were used to estimate the expression levels of all transcripts [38, 39].

After cDNA Synthesis, samples were evaluated by qRT‐PCR by C1000 Biorad Thermal Cycler. qPCR kit (#4444556; Thermo Fisher Scientific) and primers were used to evaluate the expression of the following genes: XBP1s (Hs03929085_g1; Thermo Fisher Scientific). XBP1t (Hs00231936_m1; Thermo Fisher Scientific), HSPA5 (Hs00607129_gH; Thermo Fisher Scientific), DDIT3 (Hs00358796_g1; Thermo Fisher Scientific), GAPDH (Hs02758991_g1; Thermo Fisher Scientific), CALR (Hs00189032_m1), and ATF4 (Hs00909569_g1). Additional RNA‐Sequencing analysis was performed using GSEA (Broad Institute) or IDEP [40]. RNA‐Sequencing has been deposited to GEO (GSE221526).

### 2.7 DARTS and thermal shift assays

The DARTS assay was performed according to the protocol by Lomenick et al. [41, 42]. RPMI‐8226 and ARH‐77 cells were lysed with MPER solution (Thermo Fisher Scientific) and measured with bicinchoninic acid assay (23225; Thermo Fisher Scientific) to normalize protein concentrations. The cell lysates were then incubated with DMSO or 50 μm 2P‐Im for 30 min. Cell lysates were then treated with 0.025 mg·mL^−1^ with Pronase (Roche, Basel, Switzerland) for 30 min shaking on ice. Both a protease inhibitor and a phosphatase Inhibitor (A32961; Thermo Fisher Scientific) were added to solutions and they were subsequently prepared for western blot. For Lonp1 Pronase treatment, cells were treated with 0.083, 0.05, and 0.025 mg·mL^−1^ Pronase for 15 min on ice.

Thermal shift assays (ThermoFluor) were performed as previously described [43, 44]. 10 μm of recombinant GRP78 protein (PROTP11021; Boster Biological Technology, Pleasanton, CA, USA) was incubated with 150 μm 2P‐Im controlled with 1% DMSO, and thermal shift assay was performed as previously described [45].

### 2.8 Immunoblotting

Cells were lysed with Cell Lysis solution (#9803; Cell Signaling) with a protease and phosphatase inhibitor (A32961; Thermo Fisher Scientific) according to the manufacturer's instruction. After lysis, cells were measured using bicinchoninic acid assay to determine protein concentrations, and equal amounts of proteins were mixed with Nupage LDS sample buffer (NP0007; Thermo Fisher Scientific) according to the manufacturer's instructions. Samples were loaded into SDS/PAGE gel and then transferred to PVDF membranes (Catalog #IPFL00010; Millipore, MA, USA), and incubated with blocking buffer (927‐60001; Li‐cor, Lincoln, NE, USA). Membranes were incubated with primary antibodies overnight at 4 °C, washed three times with Tris‐buffered saline with 0.05% Tween‐20, and then with the corresponding secondary antibody for 1 h at room temperature. Membranes were washed three times with Tris‐buffered saline with 0.05% Tween‐20. Finally, membranes were visualized with Li‐Cor Odyssey DLx using image studio.

### 2.9 Inhibition of the UPR in RPMI‐8226

RPMI‐8226 with deletion of the genes encoding either *PERK* or *DDIT3* were purchased (Synthego, Redwood, CA, USA) and seeded into 96‐well plates for single colonies. Gene deletions were generated by using CRISPR‐Cas9 and sgRNA (the sgRNA for Perk was ACCAUGAUUUUCAGGAUCCA and for Ddit3 was AUUUCCAGGAGGUGAAACAU). Cells were then seeded at a density of one cell per well in 96‐well plates and grown until colonies were established. Colonies were tested by western blot for knockout of target proteins. Reversal of the effects caused by eIF2a phosphorylation through ISRIB administration was performed using 0.25 μm of ISRIB for 3 h prior to a 16 h incubation with either DMSO (1%) or 2P‐Im at the specified concentrations. Cells were evaluated for cell viability using CellTiter‐Glo™. For immunoblotting and qRT‐PCR of ISRIB‐treated cells, cells were incubated with ISRIB for 3 h before treatment with 0.4 μm CDDO‐2P‐Im for 6 h. Cells were then harvested for RNA or protein.

### 2.10 Statistical analysis

The data are expressed as mean ± standard deviation (SD) except mouse data which is expressed as mean ± standard error (SEM). A two‐sided unpaired Student's *t*‐test was used to compare the statistical difference between groups. A value of *P* < 0.05 was considered significant.

## 3 Results

### 3.1 2P‐Im suppresses myeloma proliferation and induces apoptosis both alone and in combination with ixazomib

**Fig. 1:**
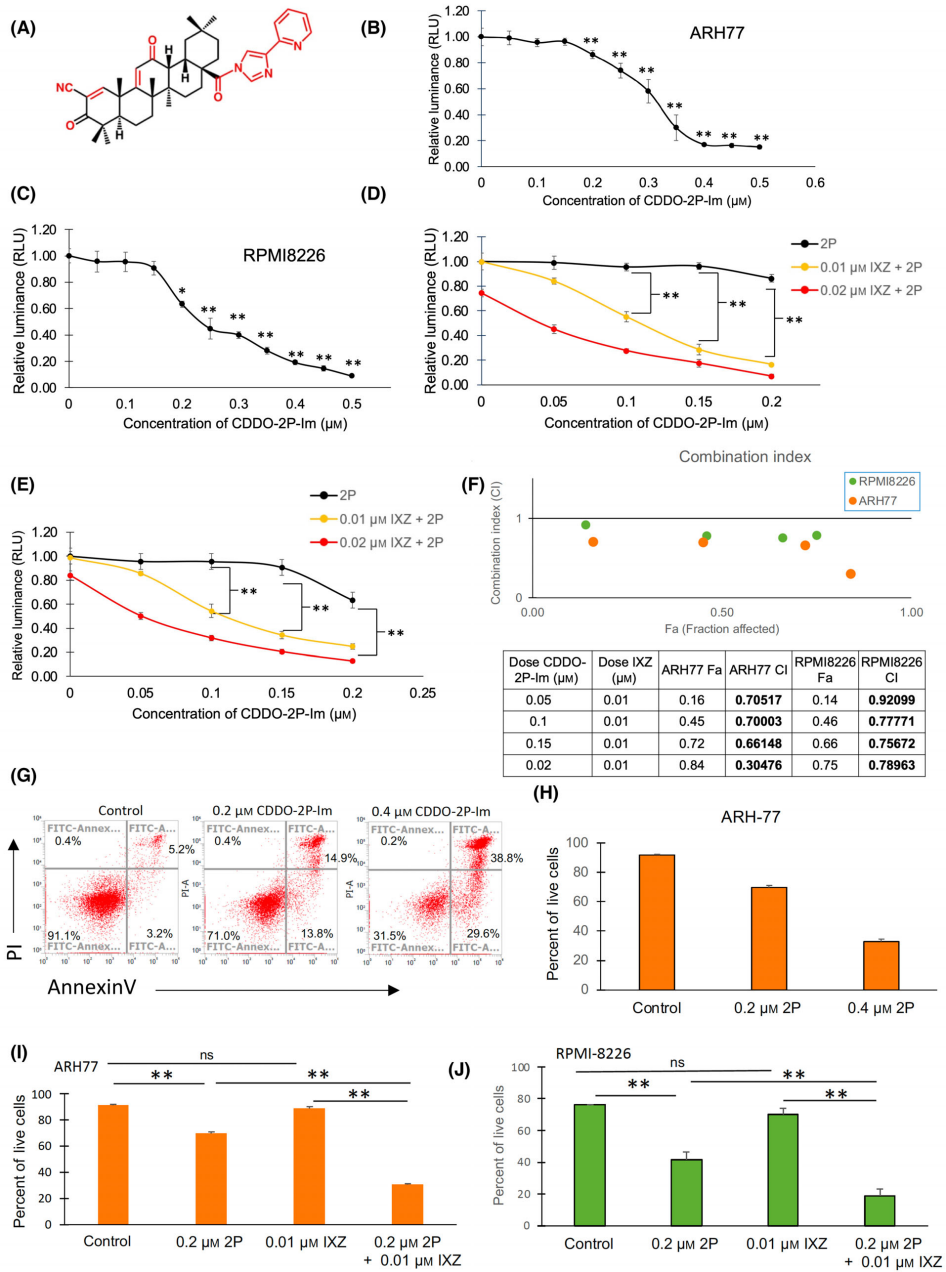
CDDO‐2P‐Im suppresses myeloma proliferation and induces apoptosis both alone and in combination with ixazomib. (A) The structure of CDDO‐2P‐Im is shown, prepared with chemdraw (PerkinElmer, Waltham, MA, USA). The red colored areas represent a modification to the oleanane backbone. (B) ARH‐77 cells and (C) RPMI‐8226 were preincubated with DMSO or CDDO‐2P‐Im for 24 h and evaluated for cell viability with CellTiter‐Glo. (D) ARH‐77 cells and (E) RPMI‐8226 cells were preincubated with DMSO, CDDO‐2P‐Im, and/or ixazomib (IXZ) for 24 h and evaluated for cell viability with CellTiter‐Glo. (F) Combination index (CI) was calculated for the combination treatment of ARH‐77 and RPMI‐8226 cells with 0.01 μm IXZ and CDDO‐2P‐Im. (G) Analysis of annexin V and propidium iodide staining of RPMI‐8226 was performed after 24 h of treatment. (H) Analysis of live ARH‐77 cells (annexin V‐/propidium iodide‐) was performed. (I) ARH‐77 and (J) RPMI‐8226 cells were treated with DMSO, CDDO‐2P‐Im, IXZ, or combination of CDDO‐2P‐Im and IXZ for 24 h before flow cytometry analysis of annexin V/propidium iodide staining. Values were given as mean ± SD. Data are representative of three independent experiments. Student *t*‐tests were performed to calculate *P* value. *P < 0.05 and **P < 0.01, compared with control. 2P, CDDO‐2P‐Im; IXZ, ixazomib.

CDDO‐2P‐Im shares the same A ring on the left side of its structure as previous SOTs but has better bioavailability properties due to its pyridine ring on the right side (see chemical structure, Fig. 1A). To evaluate the cytotoxicity of 2P‐Im, ARH‐77, and RPMI‐8226 cells were incubated with either vehicle control (1% DMSO) or various concentrations of 2P‐Im for 24 h and analyzed with CellTiter‐Glo™ for cell viability. We found a dose‐dependent inhibitory effect of 2P‐Im in both ARH‐77 and RPMI‐8226 cells at nanomolar concentrations (Fig. 1B,C). The half‐maximal inhibitory concentration (IC_50_) of 2P‐Im was 0.30 and 0.21 μm for ARH‐77 and RPMI‐8226, respectively (Fig. 1B,C). Importantly, the PI ixazomib shifts the dose–response curve of 2P‐Im significantly for both ARH‐77 and RPMI‐8226 (Fig. 1D,E). The IC_50_ of 2P‐Im in ARH‐77 with 0.01 μm of ixazomib, which does not affect cell viability alone, is reduced to 0.12 μm (Fig. 1D). Similarly, the IC_50_ of 2P‐Im in RPMI‐8226 with 0.01 μm of ixazomib is reduced to 0.11 μm (Fig. 1E). Using the combination index, we found that various combinations of 2P‐Im and 0.01 μm ixazomib were synergistic in both ARH‐77 and RPMI‐8226 cells (Fig. 1F). CDDO‐2P‐Im can also induce apoptosis equally effectively in IXZ‐resistant myeloma cells (Fig. S1A–D).

To investigate whether apoptosis was induced by 2P‐Im, we used flow cytometry to measure Annexin V/propidium iodide staining (Fig. 1G). We found that the cell viability, defined by Annexin V/propidium staining, decreases in the presence of 2P‐Im in a similar trend as that observed in the CellTiter‐Glo™ assay (Fig. 1H; Fig. S1E–G). Furthermore, we confirmed the synergistic effects between 2P‐Im and ixazomib by utilizing the Annexin V/propidium iodide staining in both ARH‐77 and RPMI‐8226 cells (Fig. 1I,J).

### 3.2 2P‐Im slows the growth of 5T33MM cells in a mouse plasmacytoma model

**Fig. 2:**
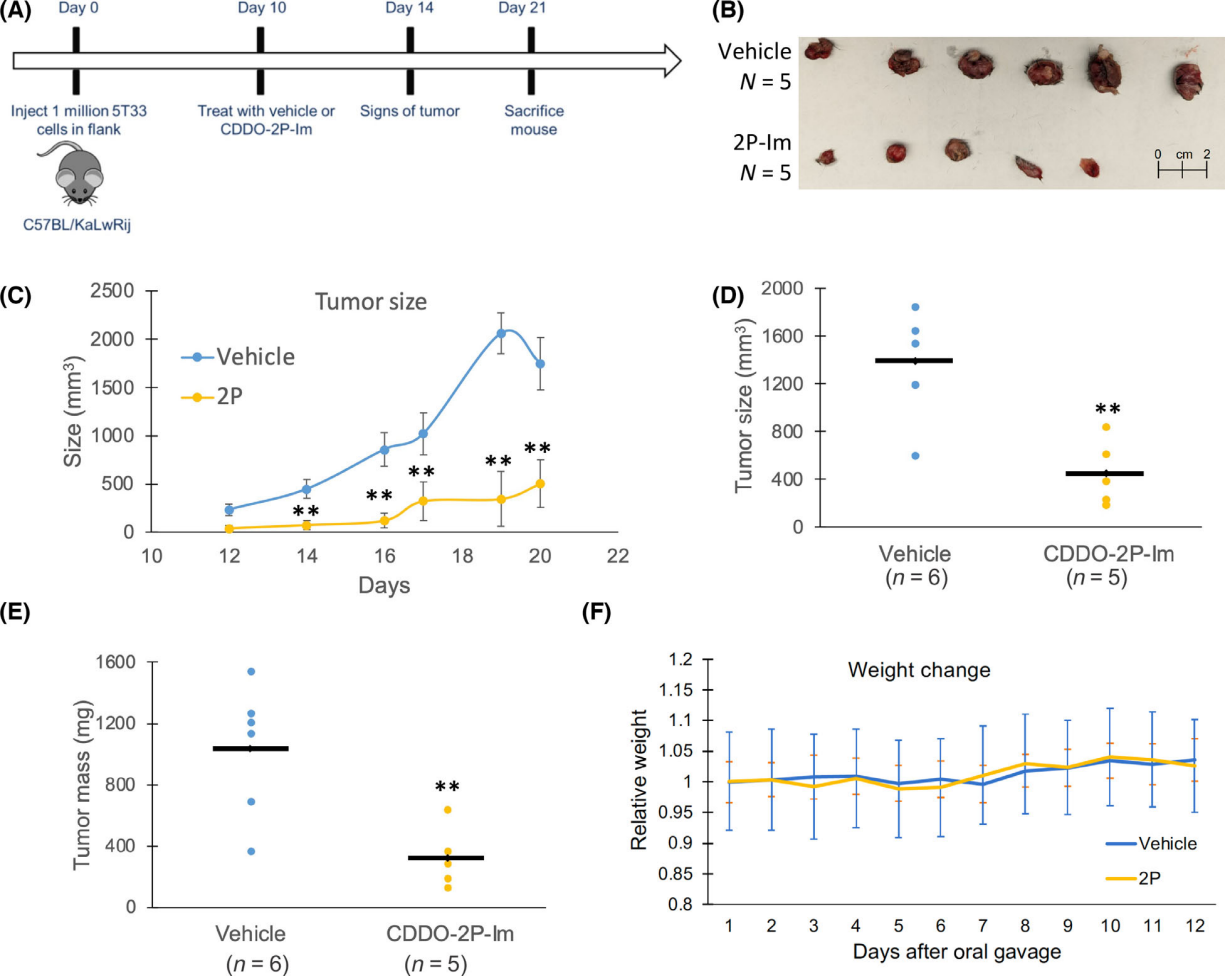
2P‐Im slows the growth of 5T33MM cells in a mouse plasmacytoma model. (A) Schematic of mouse plasmacytoma model is shown. 1 × 10^6^ 5T33 cells were injected in the flank of C57BL/KaLwRij mouse and evaluated for 21 days. Mice were treated starting day 10 with either vehicle or 24 mg·kg^−1^ CDDO‐2P‐Im by oral gavage. (B) Tumors were extracted from six vehicle‐treated and five 2P‐Im‐treated mice at the time of sacrifice and photographed. A scale bar up to 2 cm is shown for reference. (C) Tumor growth was monitored over the course of the experiment with an electronic caliper. Tumor size (D) and mass (E) were measured at the time of sacrifice. Black bars represent the mean of measurements. (F) Mice weight during treatment period was recorded. The colored line represents the relative weight while the colored bars represent the standard error for weight. The mouse experiment was performed once. Values were given as mean ± SEM. Student *t*‐tests were performed to calculate *P* value. *P < 0.05 and **P < 0.01, compared with control.

To evaluate whether 2P‐Im could suppress the *in vivo* progression of myeloma, we utilized the murine 5T33MM plasmacytoma model. In this syngeneic plasmacytoma model, 5T33MM cells were injected into the mouse flank (Fig. 2A). Mice were treated with either vehicle or 2P‐Im at day 10 and sacrificed on day 21. We observed a significant reduction in tumor size in the animals receiving 2P‐Im (24 mg·kg^−1^), when compared to animals receiving vehicle controls (Fig. 2B–D). Additionally, the tumor mass was also significantly decreased in the presence of treatment (Fig. 2E). Overall, treatment with 2P‐Im at this dose was well tolerated, as mice had nonsignificant changes in body weight compared with vehicle control (Fig. 2F). We also observed a similar trend of slowing down tumor growth in a xenograft model of ARH‐77 in NSG (NOD scid gamma) mice (Fig. S2). However, NSG mice could only tolerate a lower dose of 2P‐Im which diminished the observed effect as toxicities such as weight loss and lethargy were present at higher doses which had been observed with high doses of previous SOTs [46].

### 3.3 Transcriptomic analysis of 2P‐Im treatment reveals multiple altered pathways

**Fig. 3:**
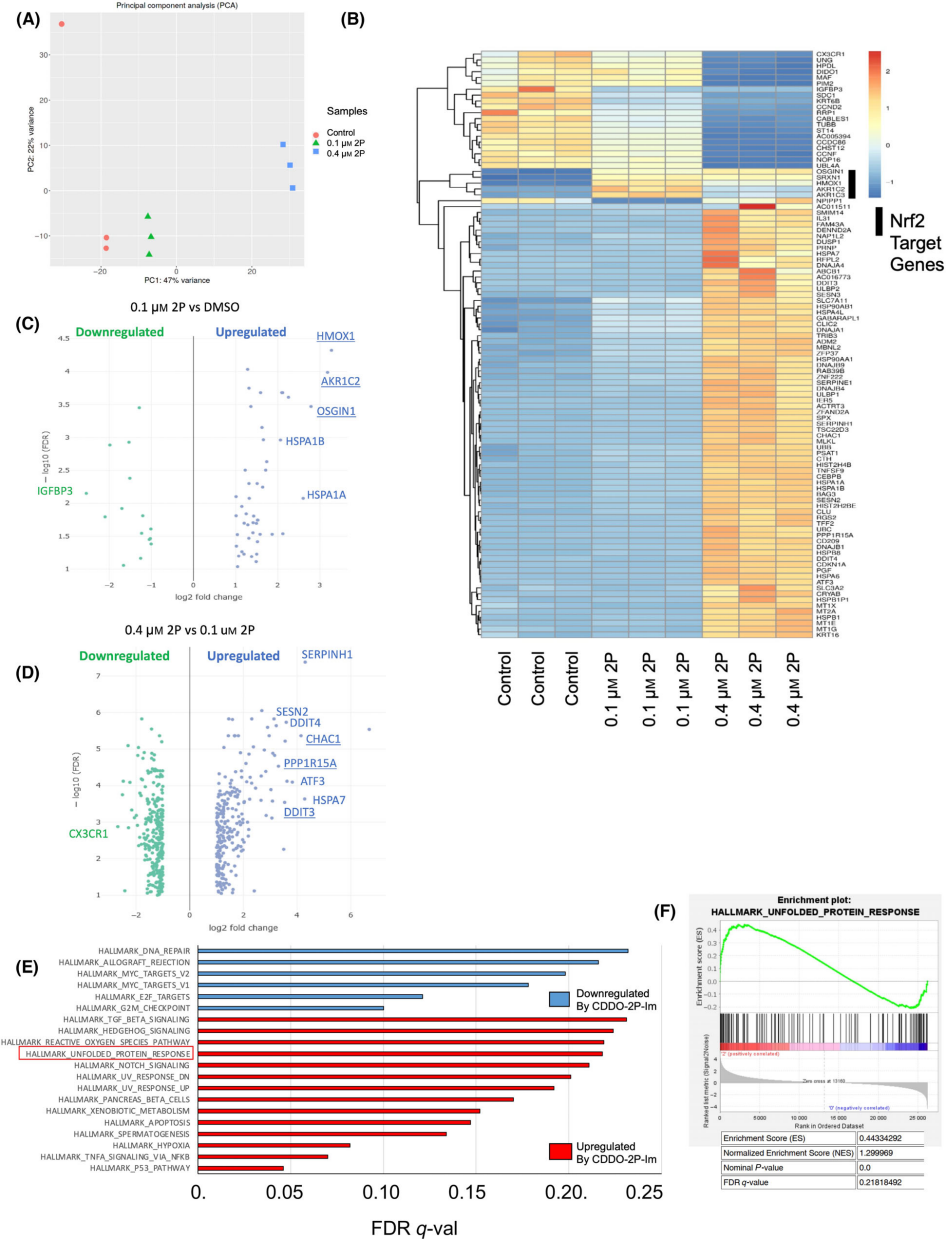
Transcriptomic analysis of 2P‐Im treatment reveals multiple altered pathways. (A) Principal component analysis of RNA‐sequencing from RPMI‐8226 with different treatments for 6 h is plotted in a two‐dimension grid. (B) Heatmap of gene expression changes between the different treatments is shown. (C) Volcano plot of gene expression changes between 0.1 μm CDDO‐2P‐Im treatment and DMSO treatment is shown with most significant gene changes labeled. (D) Volcano plot of gene expression changes between 0.4 μm CDDO‐2P‐Im treatment and 0.1 μm CDDO‐2P‐Im is shown with most significant gene changes labeled. (E) Pathway analysis of most significant hallmark pathways using GSEA of 0.4 μm CDDO‐2P‐Im and DMSO treatment is plotted. (F) The enrichment plot of the hallmark UPR comparing 0.4 μm CDDO‐2P‐Im and DMSO treatment is shown. RNA‐Sequencing was performed once. 2P, CDDO‐2P‐Im.

To understand the molecular pathways affected by 2P‐Im, we performed RNA‐sequencing of RPMI‐8226 cells after treatment with control or 2P‐Im. We observed consistent changes by 2P‐Im as demonstrated by the two‐dimensional principal component analysis (PCA) visualization of the RNA‐Sequencing data (Fig. 3A). A heatmap of gene expression changes also showcases a multitude of genes that are either downregulated or upregulated with 2P‐Im treatment (Fig. 3B). When we used volcano plots to discern differences between vehicle control and 0.1 μm 2P‐Im, we observed a significant upregulation in genes such as *HMOX1*, *AKR1C2*, and *OSGIN1*, which are known Nuclear factor E2‐related factor 2 (Nrf2) target genes involved in both antioxidant and ROS signaling (underlined in Fig. 3C) [47]. Additionally, we observed upregulation in heat shock proteins such as *HSPA1A* and *HSPA1B*. However, from our previous cytotoxicity assays, we know that 0.1 μm 2P‐Im causes insignificant changes to MM cell viability, suggesting these gene expression changes may not be directly related to the apoptosis seen at higher concentrations. Previous studies have shown that SOTs are inhibitors of Kelch‐like ECH‐associated protein 1 (KEAP1) and can activate Nrf2 signaling [8], but it appears the activation of this pathway happens at lower concentrations of SOTs and may not be related to 2P‐Im effects on MM cell viability. In fact, most Nrf2 target genes such as *HMOX1*, *AKR1C2*, and *OSGIN1*, have a higher expression at 0.1 μm than 0.4 μm 2P‐Im (Fig. 3B).

Compared with the effects observed at low concentrations of 2P‐Im in MM, concentrations of 0.4 μm 2P‐Im induced far more changes with a number of the most significantly upregulated genes being associated with the UPR such as *CHAC1*, *DDIT3*, and *PPP1R15A* (underlined in Fig. 3D) [48, 49]. These data suggested the UPR was being distinctly activated at higher concentrations of 2P‐Im treatment but not at lower concentrations. Indeed, while a number of pathways were modulated by 2P‐Im in MM cells (Fig. 3E; Fig. S3 and Tables S1–S6), the UPR pathway was uniquely enriched at higher concentrations of 2P‐Im (Fig. 3F). While we observed small increases in ROS signaling at these concentrations, they did not appear to correlate with the degree of MM cell apoptosis (Fig. S4). In sum, the data point to modulation of the UPR by 2P‐Im as a likely mechanism mediating the induction of MM apoptosis and underlying the observed synergy with PIs.

### 3.4 UPR genes are upregulated by 2P‐Im in MM, and enhanced by co‐treatment with ixazomib

**Fig. 4:**
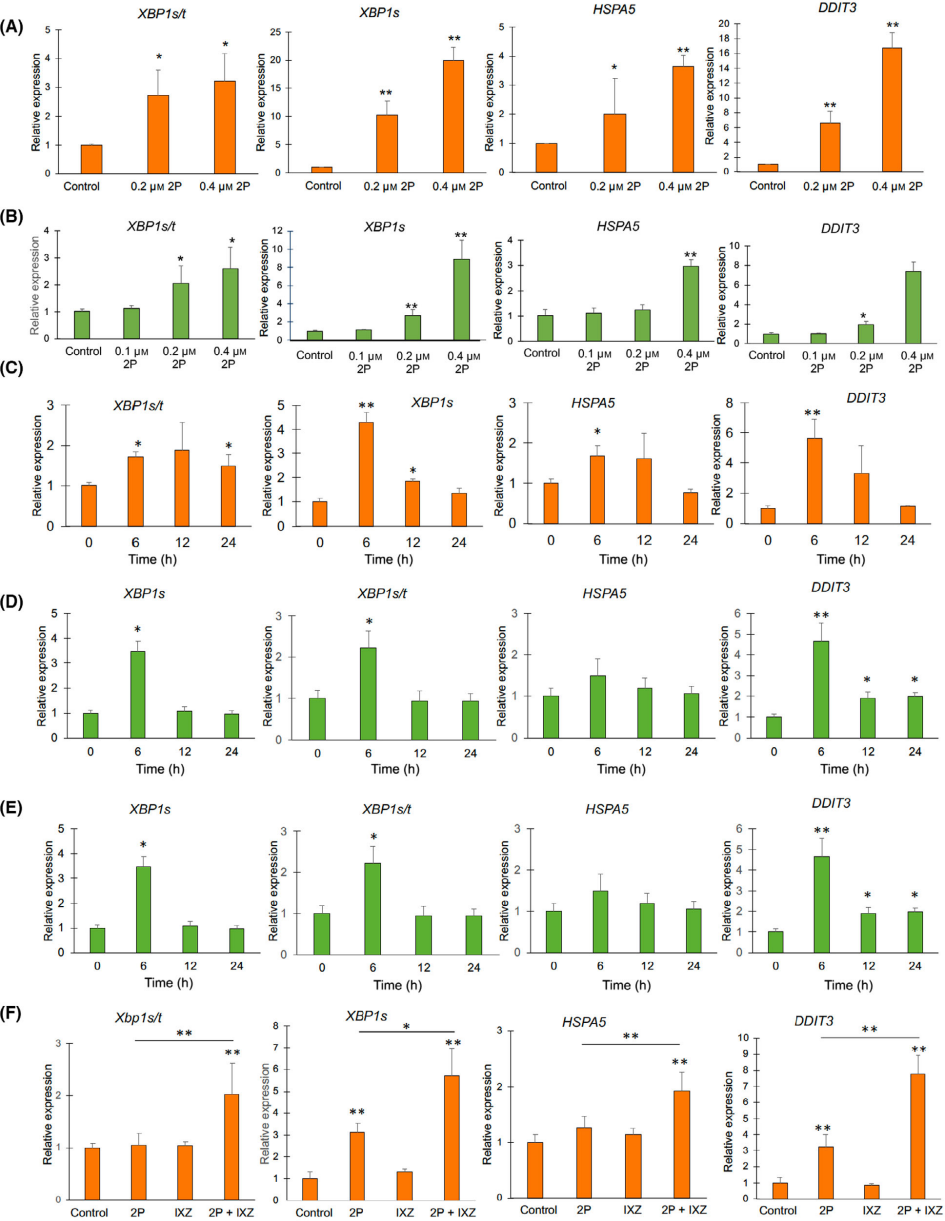
UPR genes are upregulated by CDDO‐2P‐Im in MM, and enhanced by co‐treatment with ixazomib (A, B) UPR gene expression following 6 h of CDDO‐2P‐Im treatment using qRT‐PCR of ARH‐77 and RPMI‐8226 cells show activation of the UPR. (C, D) UPR gene expression of ARH‐77 and RPMI‐8226 cells after 6, 12, and 24 h of 0.2 μm CDDO‐2P‐Im treatment. ARH‐77 (E) and RPMI‐8226 (F) were incubated with DMSO, 0.2 μm CDDO‐2P‐Im, 0.02 μm IXZ, or a combination of 0.2 μm CDDO‐2P‐Im and 0.02 μm IXZ for 12 h. RNA was extracted and UPR gene expression was measured by qRT‐PCR. Graphs showing ARH‐77 data is colored orange while RPMI‐8226 data is colored green. Values were given as mean ± SD. Data are representative of three independent experiments. All qRT‐PCR values were normalized to GAPDH. Student *t*‐tests were performed to calculate *P* value. *P < 0.05 and **P < 0.01, compared with control. 2P, CDDO‐2P‐Im; IXZ, ixazomib.

Using our RNA‐Seq data, we took a comprehensive look at changes in UPR pathway gene expression. We found that only MM exposure to 0.4 μm 2P‐Im and not to 0.1 μm 2P‐Im increased levels of X‐box binding protein 1 (XBP1) splicing, increased *ERN1* (IRE1α) mRNA expression, *HSPA5* (GRP78) mRNA expression, and *DDIT3* (CHOP) mRNA expression which indicates the UPR is activated (Fig. S3F). These data support the hypothesis that the activation of the UPR associates with induction of MM apoptosis by 2P‐Im. Additional data support this conclusion as we observed a dose‐dependent modulation of UPR gene expression at concentrations between 0.1 and 0.4 μm 2P‐Im in RPMI‐8226 cells (Fig. 4B). We found the same trend in ARH‐77 cells exposed to 2P‐Im, which again correlates with a decrease in cell viability seen at these concentrations (Fig. 4C). We also tested whether the activation of the UPR was time‐dependent and found UPR activation by 0.2 μm 2P‐Im was transient instead of constant (Fig. 4D,E). To see whether enhanced activation of the UPR pathway could explain the synergy seen between 2P‐Im and the PI, ixazomib, we treated cells for 12 h and investigated changes in UPR activation. The combination of 2P‐Im plus ixazomib significantly increased UPR pathway activation relative to 0.2 μm 2P‐Im alone (Fig. 4F,G). These data suggest that MM cells only transiently activate the UPR at lower doses of 2P‐Im, returning to homeostasis, whereas MM cells exposed to the combination of 2P‐Im plus a PI are more susceptible to proteomic stress and more likely to undergo apoptosis.

### 3.5 2P‐Im binds GRP78 and activates the PERK and IRE1α branches of the UPR in a dose‐dependent manner

**Fig. 5:**
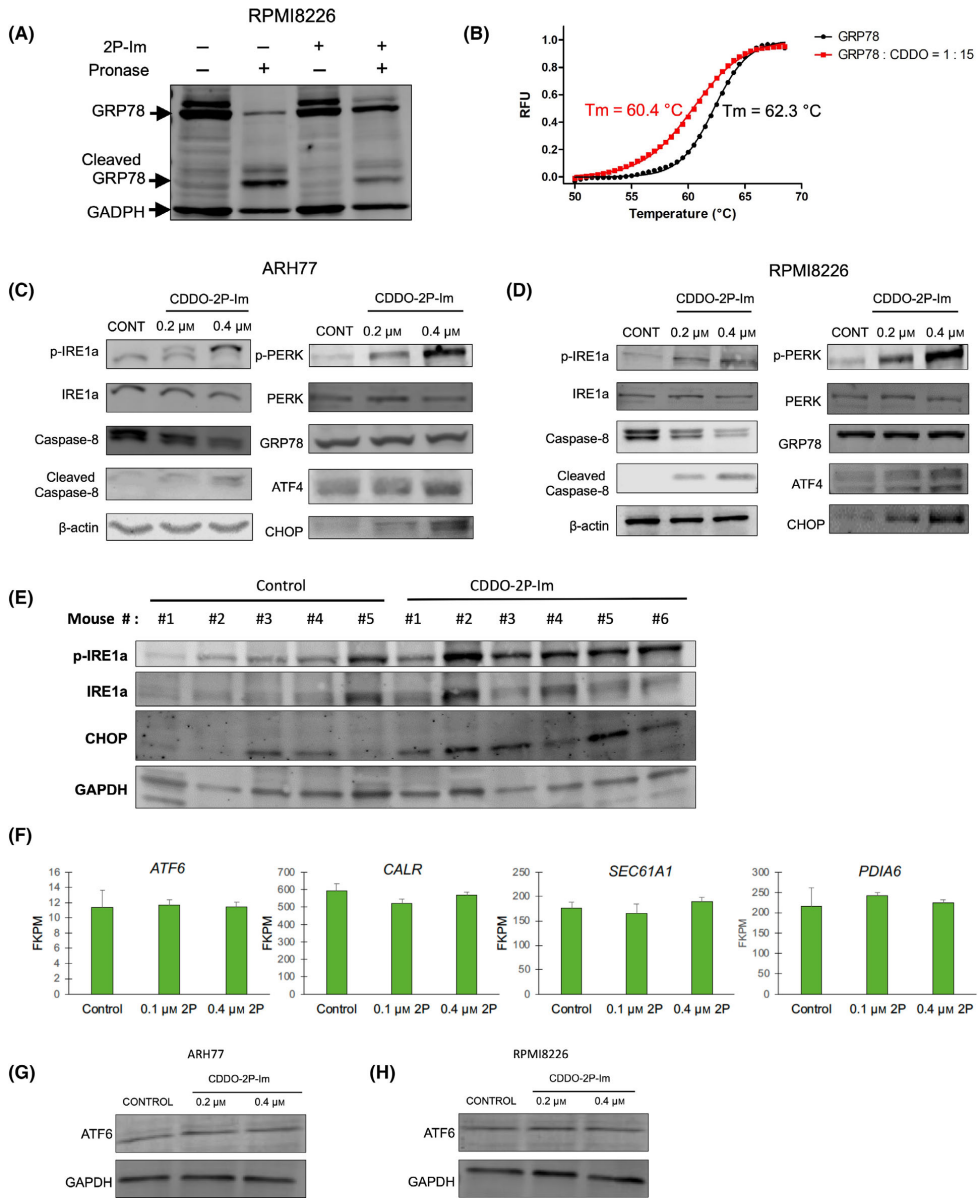
CDDO‐2P‐Im binds GRP78 and activates the PERK and IRE1α branches of the UPR in a dose‐dependent manner. (A) Cell lysate of RPMI‐8226 was incubated with DMSO or CDDO‐2P‐Im for 30 min. Lysates were then subjected to 0.025 mg·mL^−1^ pronase for 30 min at 4 °C, followed by SDS/PAGE and western blot using antibodies against GRP78 and GAPDH. CDDO‐2P‐Im protects GRP78 from pronase cleavage compared with DMSO control. (B) Thermal shift assay was performed on recombinant GRP78 treated with control (DMSO) or CDDO‐2P‐Im. Relative fluorescent units (RFU) were measured by a SpectraMax Paradigm plate reader. The melting temperature (Tm) has a shift of −1.9 °C with the treatment of CDDO‐2P‐Im. (C, D) ARH‐77 and RPMI‐8226 cells were incubated with CDDO‐2P‐Im for 6 h. Cells were lysed and samples were prepared for SDS/PAGE. Western blot of UPR signaling proteins and downstream targets were performed. (E) 5T33 tumors were extracted from mice 15 days after tumor injection. Mice were treated once with either vehicle or 24 mg·kg^−1^ CDDO‐2P‐Im for 12 h prior to extraction. Samples were immunoblotted for UPR proteins. (F) RNA‐Sequencing data for ATF6 regulated genes showed no differences between Control, 0.1 μm CDDO‐2P‐Im, and 0.4 μm CDDO‐2P‐Im in RPMI‐8226 cells. (G, H) Western blot of ATF6 in ARH‐77 and RPMI‐8226 show no changes at 6 h with the treatment of CDDO‐2P‐Im. Values were given as mean ± SD. Fragments Per Kilobase of transcript per Million mapped reads is abbreviated as FPKM. Western blot and DARTS data are representatives of at least three independent experiments for ARH‐77 cells and RPMI‐8226 cells. Thermal shift assay was performed three independent times and representative data was shown. Western blot of 5T33 tumors was performed once. RNA‐Sequencing was performed once. Student *t*‐tests were performed to calculate *P* value. 2P‐Im, CDDO‐2P‐Im; CDDO, CDDO‐2P‐Im.

The data presented thus far indicate 2P‐Im directly targets an upstream regulator of the UPR. One potential target is GRP78, as GRP78 is a key sensor of ER stress, retaining IRE1α, PERK, and ATF6 in the ER lumen to prevent the activation of corresponding downstream substrates under physiological conditions. The potential for GRP78 to serve as a target of 2P‐Im is suggested by recent studies describing GRP78 as a target protein of natural pentacyclic triterpenoids [50, 51]. To determine whether 2P‐Im, and potentially other SOTs, can bind to GRP78, we performed a DARTS assay, which is a well‐defined and validated strategy to investigate the binding of drug to target protein through proteolytic protection after drug‐protein binding [52]. We first used LONP1, a known target of SOTs, to demonstrate that this strategy works with 2P‐Im (Fig. S5). Next, we found that 2P‐Im could also protect GRP78 from cleavage, suggesting that GRP78 is a target of 2P‐Im (Fig. 5A). We corroborated these data by using a thermal shift assay, which monitors drug perturbation of protein thermostability (Fig. 5B) [44]. The significance of this observation is further supported by analyses of protein expression changes in both ARH‐77 and RPMI‐8226 cells, which show activation of the PERK and IRE1α, as well as downstream target proteins such as ATF4 and CHOP with the exception of GRP78 (Fig. 5C,D). We similarly found upregulation of CHOP and increased levels of IRE1a and p‐IRE1a in 5T33 cells isolated from subcutaneous tumors of mice treated with 2P‐Im, showing that the drug can activate the UPR *in vivo* (Fig. 5E). Surprisingly, when we evaluated ATF6‐related gene expression changes, we saw no significant changes in the RNA‐sequencing data (Fig. 5F). Additionally, we observed no changes in protein levels or cleavage of ATF6 (Fig. 5G,H) suggesting this branch of the UPR is not activated at this concentration of 2P‐Im while the other two branches, PERK and IRE1α, are activated. Tunicamycin, another UPR activator, activated all three branches as compared to only two branches by CDDO‐2P‐Im (Fig. S6).

### 3.6 Inhibition of the PERK‐ATF4‐CHOP arm of the UPR partially rescues 2P‐Im‐induced apoptosis

**Fig. 6:**
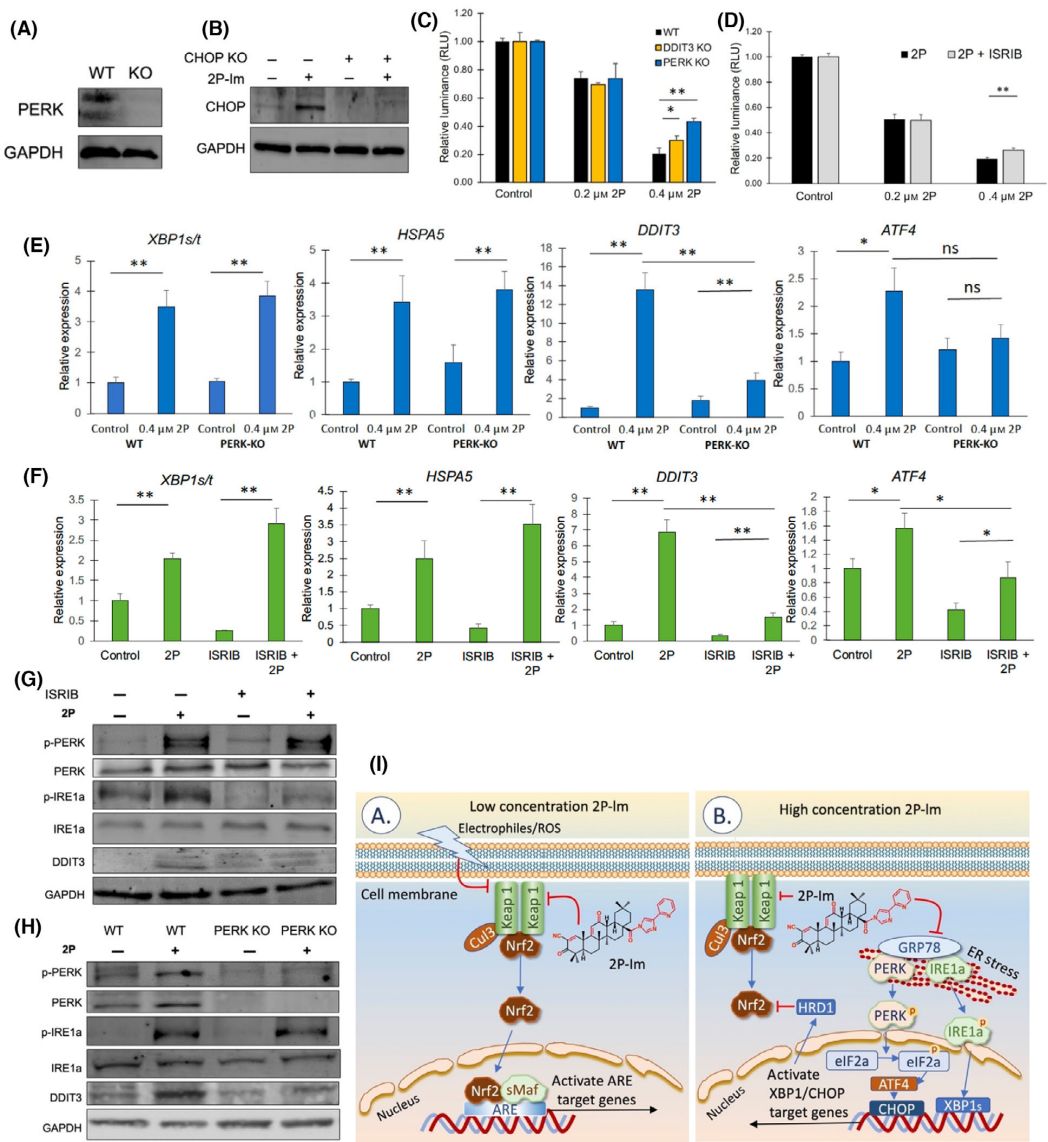
Inhibition of the PERK‐ATF4‐CHOP arm of the UPR partially rescues CDDO‐2P‐Im‐induced apoptosis. (A) Cell lysate of WT and *PERK* KO RPMI‐8226 were probed for PERK protein by western blot. (B) WT and *DDIT3* (CHOP) KO RPMI‐8226 were incubated with DMSO or CDDO‐2P‐Im for 6 h and lysed for protein analysis. Western blot of CHOP was performed to investigate the knockout of CHOP protein. (C) Cells were preincubated with DMSO or CDDO‐2P‐Im for 24 h and evaluated for cell viability by CellTiter‐Glo™. (D) Cells were preincubated with DMSO or 0.25 μm ISRIB for 3 h before treating with DMSO or CDDO‐2P‐Im for an additional 16 h. Cells were then measured for cell viability by CellTiter‐Glo™. Controls were cells that were not treated with CDDO‐2P‐im but were treated with DMSO or ISRIB where appropriate. (E–H) PERK KO and WT RPMI‐8226 cells or ARH‐77 cells pretreated with ISRIB for 3 h were incubated with DMSO control or 0.4 μm CDDO‐2P‐Im for 6 h. (E, F) Cells were extracted for RNA and qRT‐PCR was performed to investigate changes in the UPR. (G, H) In another round of experiments, cells were also extracted for protein to confirm such changes in the UPR. Values were given as mean ± SD. Student *t*‐tests were performed to calculate *P* value. *P < 0.05 and **P < 0.01, compared with control. Cell viability data are representative of three independent experiments. Western blots and qRT‐PCR data are representatives of two independent experiments. (I) We provide a working model of the actions of CDDO‐2P‐Im in cancer cells. At low concentrations (panel A), 2P‐Im binds and inhibits KEAP1, an adaptor protein for ubiquitin ligase that negatively regulates Nrf2 levels. Nrf2 will translocate to the nucleus and dimerize with small Maf proteins (sMaf) to activate the transcription of Nrf2 target genes. At higher concentrations (panel B), 2P‐Im binds GRP78/BiP, resulting in the activation of the UPR. GRP78 dissociates from its binding partners leading to the phosphorylation of PERK and IRE1α and activation of these branches of the UPR. The transcription factors, CHOP and XBP1, will cause UPR‐associated gene expression changes which when prolonged will lead to apoptosis. Interestingly, XBP1 is known to increase the expression of HRD1, a negative regulator of Nrf2, and independent of KEAP1. 2P/2P‐Im, CDDO‐2P‐Im; ISRIB, integrated stress response inhibitor; WT, wild‐type; KO, knockout.

To more definitively demonstrate the relevance of 2P‐Im activation of the UPR in the induction of MM apoptosis, we generated variants of the RPMI‐8226 cell line using CRISPR‐Cas9 to disrupt either *PERK* or *DDIT3* (CHOP) expression. Specific mutants were seeded and cell colonies were established from single cells. We confirmed colonies with either a *PERK* or *DDIT3* gene knockout by western blot (Fig. 6A,B). We then measured the cell viability of WT RPMI‐8226, and of either a PERK KO or CHOP KO derived from RPMI‐8226, in either the presence or absence of 2P‐Im treatment (Fig. 6C). We again found there was only a significant difference in MM cell viability when they were exposed to the high concentration of 2P‐Im, an effect that is partially rescued by the deletion of either PERK or CHOP. We also used a drug that reverses the effects caused by phosphorylation of eukaryotic translation initiation factor 2A (eIF2a), ISRIB, which inhibits the UPR signaling pathway downstream of PERK [53] (Fig. S7). ISRIB pretreatment of MM cells similarly reduced the effect of 2P‐Im on cell viability at the 0.4 μm concentration (Fig. 6D). We also investigated signaling changes by PERK knockout and ISRIB pretreatment when cells were treated by CDDO‐2P‐Im (Fig. 6E–H). PERK knockout and ISRIB pretreatment did not affect *XBP1* splicing but reduced *DDIT3* and *ATF4* upregulation by CDDO‐2P‐Im (Fig. 6E,F). In total, the data presented reveal GRP78 as a direct target of 2P‐Im and that this interaction result activates the UPR, which plays a significant role in 2P‐Im‐induced MM cell apoptosis, and in the synergy of 2P‐Im and PIs (Fig. 6I).

## 4 Discussion

Here we show dose‐dependent activation of the UPR by the fourth‐generation SOT, CDDO‐2P‐Im. Two independent analyses of 2P‐Im‐protein interactions reveal GRP78, a key modulator of the ER stress response, to be a direct target of 2P‐Im. In myeloma, modulation of the UPR by GRP78 promotes survival and its expression has emerged as a potential novel biomarker and therapeutic target [24]. We show the ability of 2P‐Im to activate the UPR is associated with the apoptosis of myeloma cells and 2P‐Im potentiates the myeloma response to the PI, ixazomib. These observations align with studies that show pharmacologic inhibition of GRP78‐dependent autophagy enhances the antimyeloma effect of the PI bortezomib [22]. This effect may be uniquely important in MM as myeloma cells have a lower threshold for PI‐induced UPR induction and ER stress‐induced apoptosis because they constitutively express ER stress survival factors to function as secretory cells [23]. The clinical significance of this pathway is further highlighted by recent studies demonstrating that increased activity of all three arms of the UPR is an invariable feature of both bortezomib‐ and carfilzomib‐resistant myeloma cells [54].

CDDO‐2P‐Im is a first‐in‐class pyridyl derivative of the multifunctional synthetic triterpenoid CDDO‐Imidazolide (1‐[2‐Cyano‐3,12‐dioxooleana‐1,9(11‐dien‐28‐oyl)] imidazole, ‘CDDO‐Im’) [7], and has improved plasma stability and pharmacokinetic (PK) profiles relative to CDDO‐Im. CDDO‐Im is one of the most potent known synthetic triterpenoids and has unique activities and molecular targets beyond those of its related analog, CDDO‐methyl ester (bardoxolone methyl) [55, 56], which is in current clinical trials in patients with chronic kidney disease [57, 58]. Both CDDO‐Im and 2P‐Im represent new innovative approaches to the development of drugs to be used for cancer therapy [55]. There are numerous published articles on the ability of CDDO‐Im to either treat or prevent many types of cancer in experimental animals [7, 55, 59, 60]. Proteomic and other analyses have shown that CDDO‐Im interacts with several critical networks in the cell, including those that regulate apoptosis, [14] response to oxidative stress, DNA checkpoints, and inflammation [10, 12]. All of these interactions are germane to the treatment of MM. However, CDDO‐Im has not been used clinically because of its relatively poor pharmacokinetics (PK) and its poor stability in human plasma. Therefore, the development of 2P‐Im, and a family of related pyridyl derivatives of CDDO‐Im, creates the opportunity to advance a new generation of SOTs toward clinical application.

The introduction of a second functional (imidazolide) group at Carbon‐28 of the CDDO skeleton confers biological activities that are unique to the imidazolides [56]. These unique activities are not shared by monofunctional relatives, such as CDDO‐methyl ester. Moreover, with respect to innovation at the molecular level, CDDO‐Im and 2P‐Im represent a new class of drugs that do not act by classical, single ‘lock and key’ mechanisms [10, 59]. Rather, these SOTs interact with common hydrophobic protein motifs [61, 62, 63, 64] that are found in many networks in many places in the cell and are physiologically integrated to achieve an overall coordinated effect.

There are several known protein targets of the SOTs including KEAP1, NF‐κB, IKKβ, STAT3, and LONP1 [61, 65, 66, 67, 68]. However, it would be difficult to attribute the broad spectrum of anti‐cancer effects to any of one these targets. LONP1 is an interesting target to consider as it is essential for cell survival and has emerged as a putative target for cancer therapy [69], and has also been implicated in resistance to PIs in myeloma [70]. However, we observed no direct changes in mitochondria oxygen consumption immediately after MM cells were treated with 2P‐Im (data not shown).

There is a growing interest in the UPR as a target for cancer therapeutics, especially in myeloma, where cells are more susceptible to proteomic stress [20, 23, 25]. Both PIs and histone deacetylation inhibitors have been shown to take advantage of ER stress in myeloma cells [71]. However, higher levels of UPR have also been associated with more malignant and drug‐resistant cancers [72, 73]. This phenotype may be attributed to the pro‐survival functions of GRP78 and the UPR, which would help cancer cells cope with the stressful tumor microenvironment [74]. However, prolonged activation of the UPR can also lead to apoptosis, suggesting a tractable strategy to exploit this pathway in cancer.

The inhibition of GRP78 by 2P‐Im leads to activation of the UPR, and prolonged activation leads to apoptosis through pro‐apoptotic CHOP signaling. Other inhibitors of GRP78 have exhibited similar effects in other cancers [75, 76]. Many pentacyclic triterpenoids have been described to activate the UPR in cancers leading to eventual apoptosis [50, 51]. Interestingly, previous studies of CDDO‐Me and other natural pentacyclic triterpenoids have shown activation of the PERK and IRE1α branches of the UPR, but none have commented on ATF6 activation [28, 50, 77, 78]. In our study, we show that both RNA and protein levels of ATF6 are unaffected by 2P‐Im, which is a surprising and interesting result. We have also confirmed this selective activation of PERK and IRE1α, but not of ATF6 is not unique to MM but applies to other cancer cells treated with 2P‐Im (data not shown). Because ATF6 transcriptional changes require translocation followed by cleavage, this process may be inhibited by 2P‐Im or the treatment conditions leading to its inability to activate. Of particular interest, PERK activation can lead to the phosphorylation of eIF2a which will lead to selective ATF4 translation [79]. ATF4 can increase levels of CHOP, a pro‐apoptotic transcription factor, which will drive apoptosis [80]. This PERK‐ATF4‐CHOP signaling axis appears to be particularly important to the apoptosis induced by the UPR including by SOTs [77, 81].

Furthermore, natural pentacyclic triterpenoids have been shown to bind GRP78 with the binding site of celastrol, confirmed to be cysteine 41 of GRP78, by click chemistry pull‐down [8, 51]. Based on the previously described electrophilic properties and cysteine reactivity of the SOTs, it is logical to speculate that 2P‐Im binds cysteine 41 of GRP78, but this remains to be confirmed. Given that cysteine 41 is a key amino acid that can be modulated to affect the activity of GRP78, this type of protein–small molecule interaction between 2P‐Im and GRP78 could lead to activation of the UPR pathway without directly causing massive proteomic stress in the rest of the cell [82].

## 5 Conclusions

Our results demonstrate that CDDO‐2P‐Im, and likely other related SOTs, can bind GRP78, leading to activation of the UPR in myeloma cells. Prolonged activation of the UPR through high concentrations of SOTs or combinations of SOTs and PIs can lead to apoptosis. Importantly, the data we provide above describe a bi‐modal activity of 2P‐Im. At low concentrations, 2P‐Im principally activates Nrf2 target gene expression, by binding to KEAP1 and promoting its degradation (Fig. 6I, panel A), an attribute shared by its predecessor (CDDO‐Im) and earlier generation SOTs [8]. However, at higher concentrations, 2P‐Im leads to UPR activation through binding to GRP78, leading to downstream activation of ATF4/CHOP and XBP1s target genes (Fig. 6I, panel B). The latter may play an important role in the decreased expression of Nrf2 target genes through induction of HRD1 expression [83, 84, 85]. In summary, these data not only demonstrate the therapeutic potential of this class of compounds that warrants their continued development but also highlights their potential to act synergistically with existing agents to overcome therapy resistance.

## Conflict of interest

MBS was the founder of Triterpenoid Therapeutics, Inc.

## Author contributions

GL and JJL conceived and designed the project. GL and JJL proposed specific experiments to address the hypotheses. GL, TC, VA, B‐GK, and SL performed experiments. KA, NS, and EHH supported the project through the maintenance of mouse colonies, lab equipment, coordinating the purchase of needed supplies and reagents, and training of students participating in the project; GL, MBS, TSX, and JJL analyzed the data. GL and JJL wrote the manuscript.

### Peer review

The peer review history for this article is available at https://www.webofscience.com/api/gateway/wos/peer‐review/10.1002/1878‐0261.13447.

## Supporting information


**Fig. S1.** CDDO‐2P‐Im slows proliferation and induces apoptosis in RPMI‐8226 cells and IXZ‐resistant RPMI‐8226 cells.
**Fig. S2.** CDDO‐2P‐Im slows growth tumor growth in a tumor xenograft mouse model.
**Fig. S3.** CDDO‐2P‐Im affects multiple pathways to reduce proliferation and induce apoptosis of RPMI‐8226 myeloma cells.
**Fig. S4.** High CDDO‐2P‐Im treatment slightly elevated ROS levels.
**Fig. S5.** DARTS assay confirms LonP1 is a binding target of CDDO‐2P‐Im.
**Fig. S6.** CDDO‐2P‐Im and tunicamycin activate UPR in myeloma cells.
**Fig. S7.** ISRIB treatment does not affect cell viability.

**Table S1.** Upregulated pathways of 0.1 μM 2P‐Im treatment compared with control treatment.
**Table S2.** Downregulated pathways of 0.1 μM 2P‐Im treatment compared with control treatment.
**Table S3.** Upregulated pathways of 0.4 μM 2P‐Im treatment compared with control treatment.
**Table S4.** Downregulated pathways of 0.4 μM 2P‐Im treatment compared with control treatment.
**Table S5.** Differentially expressed genes of 0.1 μM 2P‐Im treatment compared with control treatment.
**Table S6.** Differentially expressed genes of 0.4 μM 2P‐Im treatment compared with control treatment.

## Data Availability

The data that support the findings of this study are available from the corresponding authors (jjl26@case.edu; gxl263@case.edu) upon reasonable request. The mRNA‐sequencing data have been deposited and can be accessed from the Gene Expression Omnibus (GSE221526, GEO; https://www.ncbi.nlm.nih.gov/geo/).
