# Supplementary material for: The synthetic oleanane triterpenoid CDDO‐2P‐Im binds GRP78/BiP to induce unfolded protein response‐mediated apoptosis in myeloma
Source: Mol Oncol. 2023 Jun 13;17(12):2526–45. doi: 10.1002/1878-0261.13447 (PMC10701780; doi:10.1002/1878-0261.13447)

Supplemental Figure 1

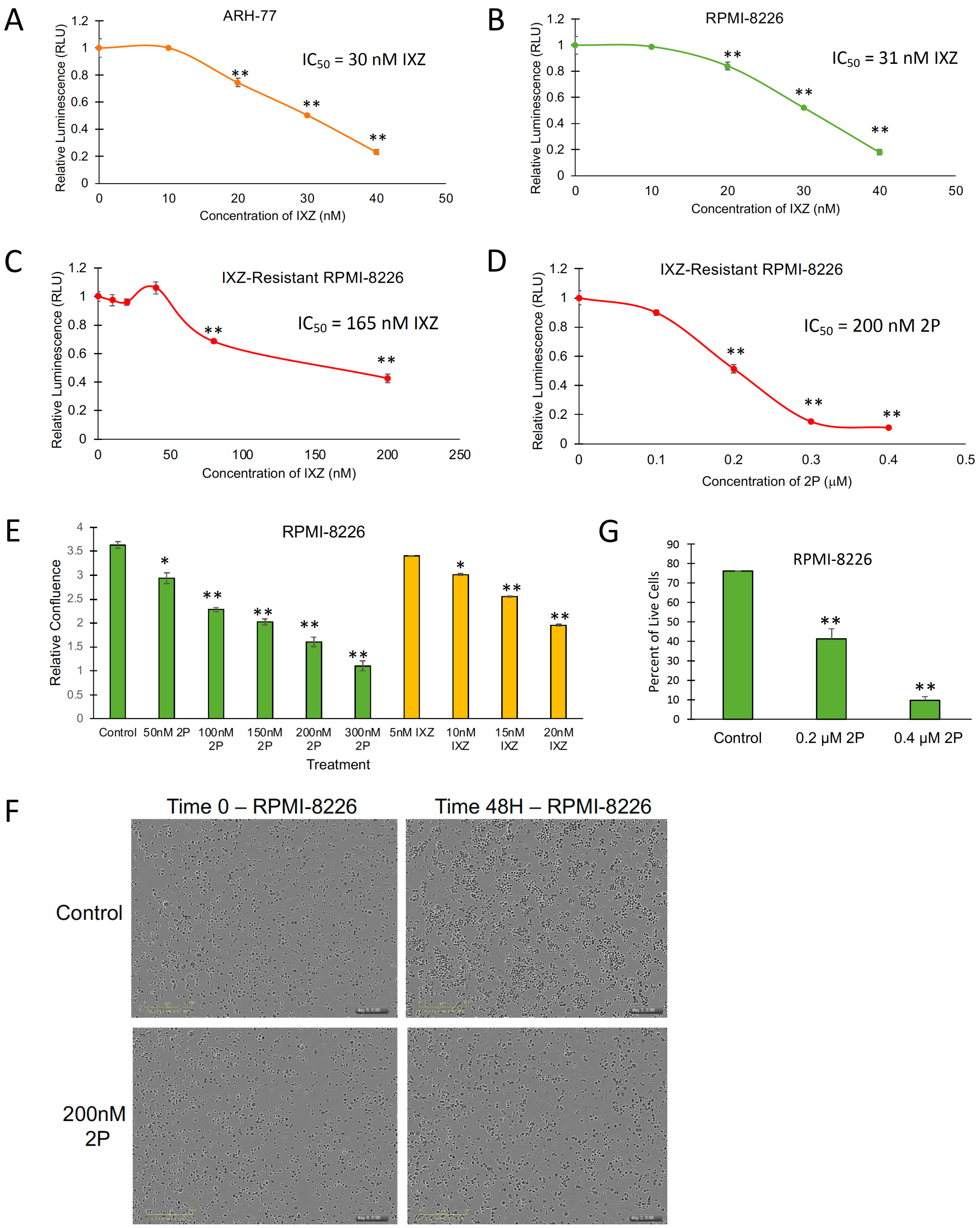

Supplemental Figure 2

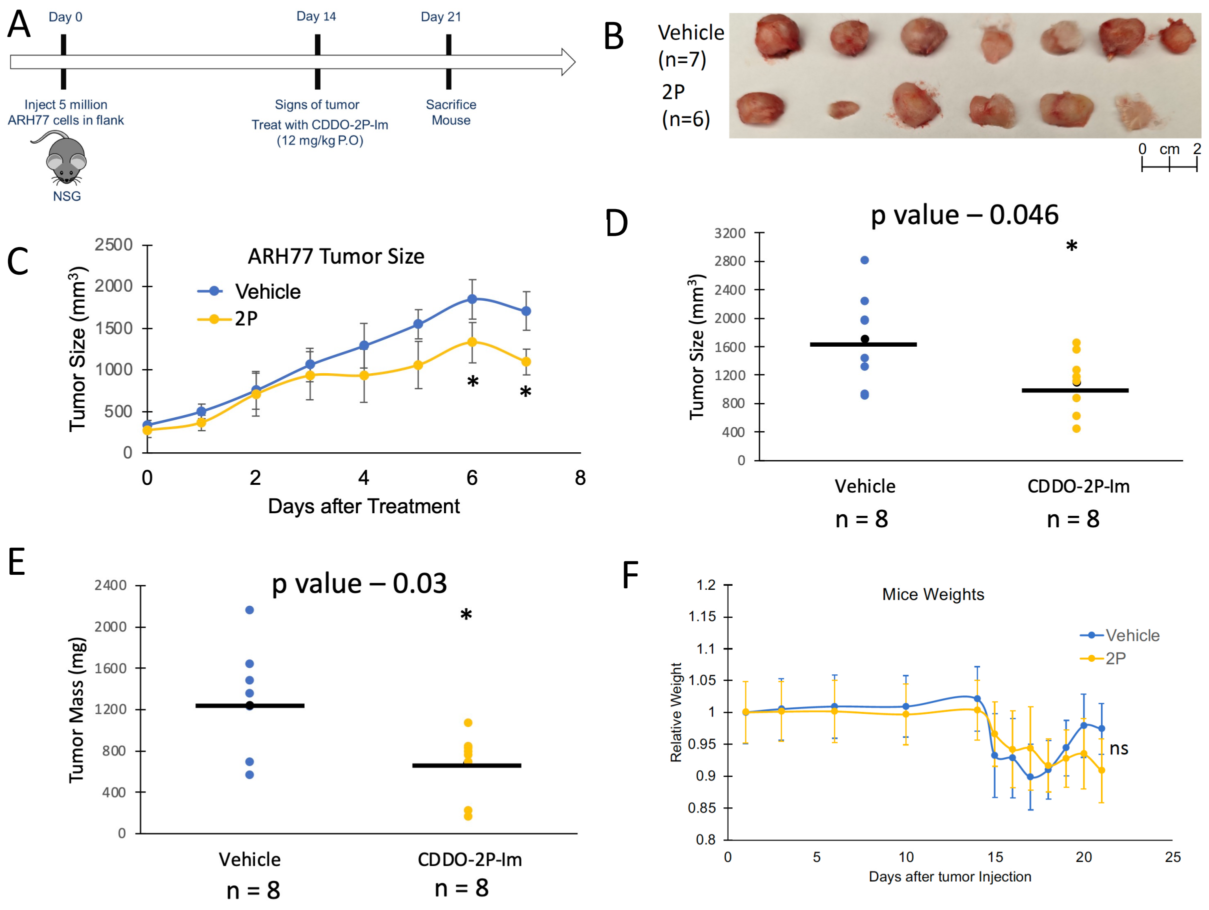

Supplemental Figure 3

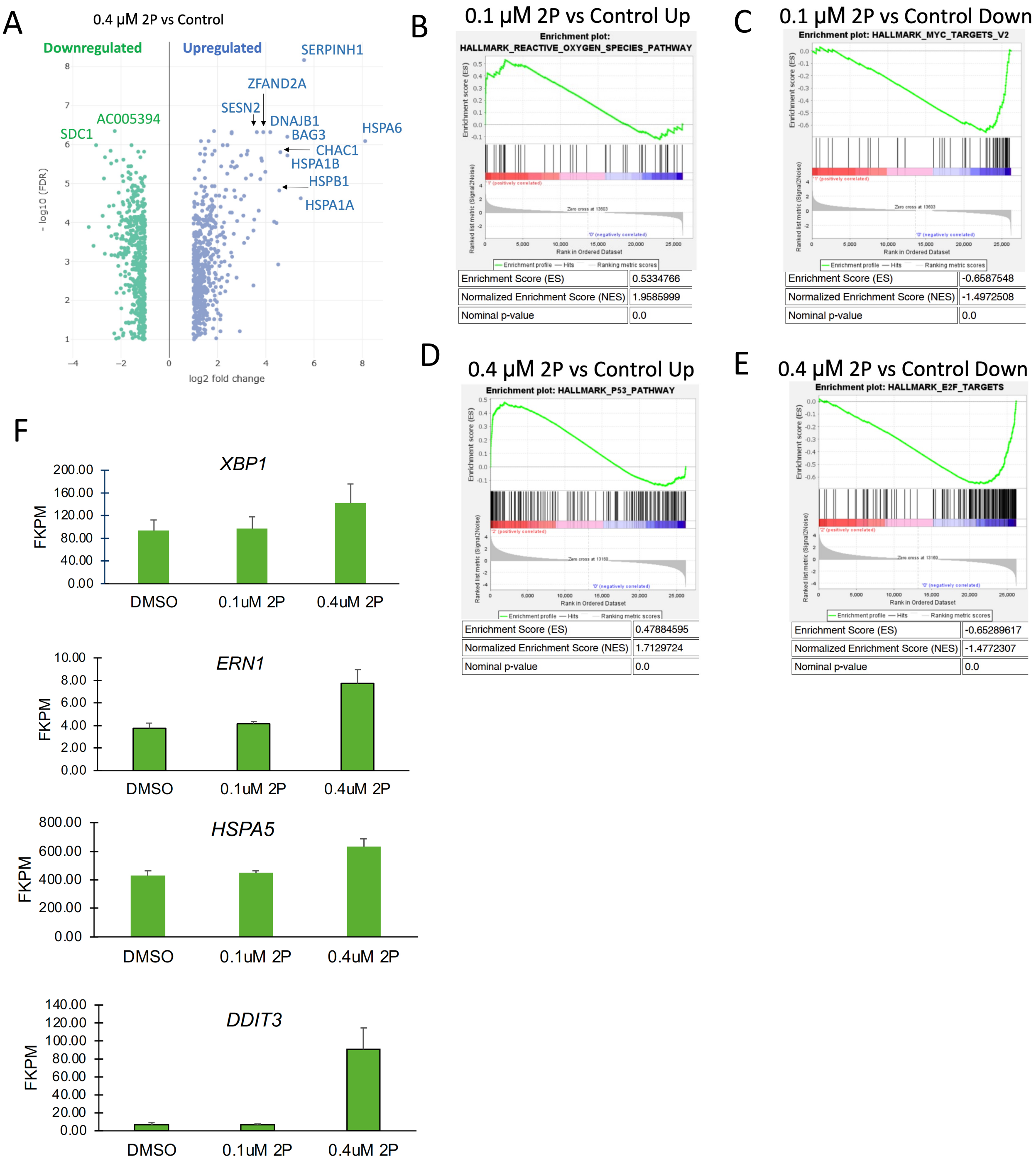

Supplemental Figure 4

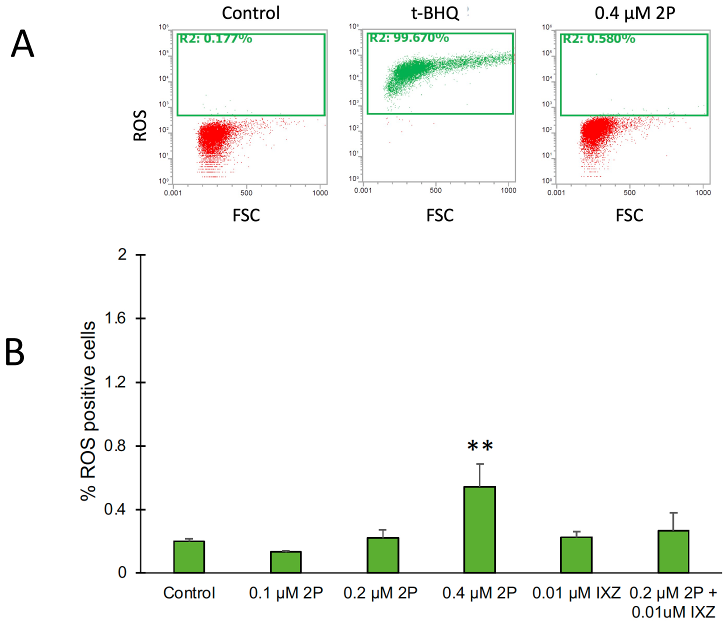

Supplemental Figure 5

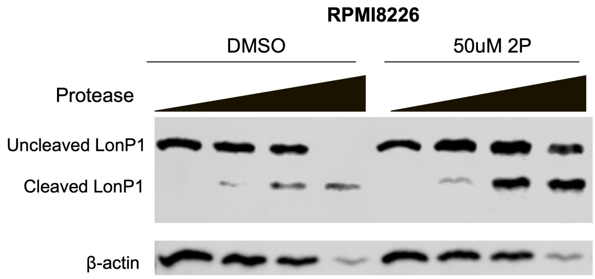

Supplemental Figure 6

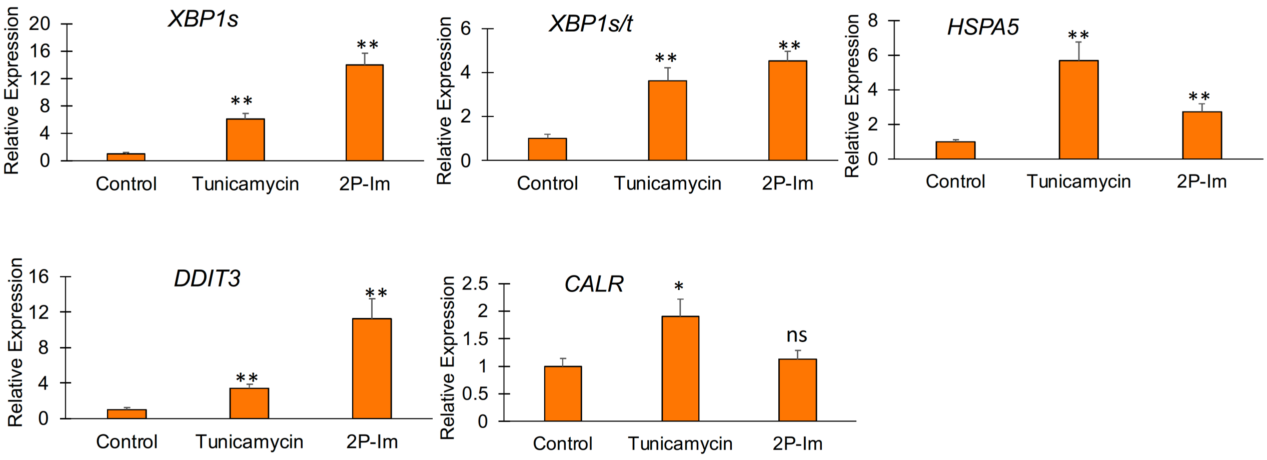

Supplemental Figure 7

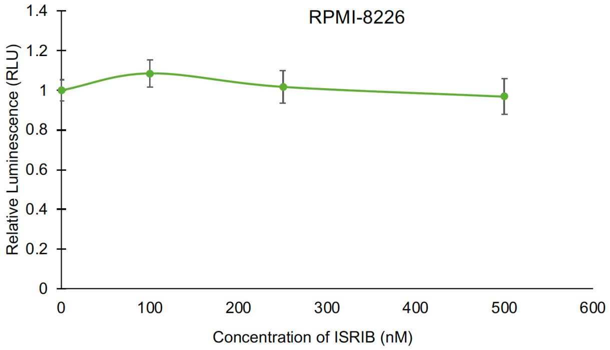

Supplement: Supplementary file 1 — Fig. S1. CDDO‐2P‐Im slows proliferation and induces apoptosis in RPMI‐8226 cells and IXZ‐resistant RPMI‐8226 cells. Fig. S2. CDDO‐2P‐Im slows growth tumor growth in a tumor xenograft mouse model. Fig. S3. CDDO‐2P‐Im affects multiple pathways to reduce proliferation and induce apoptosis of RPMI‐8226 myeloma cells. Fig. S4. High CDDO‐2P‐Im treatment slightly elevated ROS levels. Fig. S5. DARTS assay confirms LonP1 is a binding target of CDDO‐2P‐Im. Fig. S6. CDDO‐2P‐Im and tunicamycin activate UPR in myeloma cells. Fig. S7. ISRIB treatment does not affect cell viability. [file MOL2-17-2526-s002.zip › mol213447-sup-0001-FigureS1-S7.pdf]
