# Supplementary material for: The synthetic oleanane triterpenoid CDDO‐2P‐Im binds GRP78/BiP to induce unfolded protein response‐mediated apoptosis in myeloma
Source: Mol Oncol. 2023 Jun 13;17(12):2526–45. doi: 10.1002/1878-0261.13447 (PMC10701780; doi:10.1002/1878-0261.13447)
Supplement: Supplementary file 1 — Fig. S1. CDDO‐2P‐Im slows proliferation and induces apoptosis in RPMI‐8226 cells and IXZ‐resistant RPMI‐8226 cells. Fig. S2. CDDO‐2P‐Im slows growth tumor growth in a tumor xenograft mouse model. Fig. S3. CDDO‐2P‐Im affects multiple pathways to reduce proliferation and induce apoptosis of RPMI‐8226 myeloma cells. Fig. S4. High CDDO‐2P‐Im treatment slightly elevated ROS levels. Fig. S5. DARTS assay confirms LonP1 is a binding target of CDDO‐2P‐Im. Fig. S6. CDDO‐2P‐Im and tunicamycin activate UPR in myeloma cells. Fig. S7. ISRIB treatment does not affect cell viability. [file MOL2-17-2526-s002.zip › mol213447-sup-0002-Legends.pdf]

Supplemental Fig. 1.

CDDO-2P-Im slows proliferation and induces apoptosis in RPMI-8226 cells and IXZ-resistant RPMI-8226 cells. (A and B) ARH-77 and RPMI-8226 cells were treated with DMSO or IXZ for 24 hours before being evaluated for cell viability by CellTiter-Glo. (C and D) IXZ-resistant RPMI-8226 cells were treated with DMSO, IXZ, or CDDO-2P-Im for 24 hours before being evaluated for cell viability by CellTiter-Glo. (E) RPMI-8226 cells were treated with various concentrations of CDDO-2P-Im or IXZ and incubated for 48 hours in the Incucyte. Confluence of cells was measured in each well with 4 images per well to calculate the mean confluence and standard deviations. (F) A representative image from the Incucyte is displayed. A measurement scale bar of 300 μm is added for reference. (G) RPMI-8226 cells were incubated with various concentrations of CDDO-2P-Im for 24 hours and stained with annexin V/propium iodide to detect live and dead cells. The percent of live cells was quantified and graphed. Values were given as mean ± SD. * $P < 0.05$ and ** $P < 0.01$, compared with control.

Supplemental Fig. 2.

CDDO-2P-Im slows growth tumor growth in a tumor xenograft mouse model. (A) Schematic of mouse experiment and timeline is shown. 5×10^6 ARH-77 cells were injected in the flank of NOD scid gamma (NSG) mice. Mice were treated on day 14 when tumors were present in mice. Mice were treated with vehicle or 12 mg/kg CDDO-2P-Im through oral gavage five times over the course of 7 days. Mice were sacrificed at day 21. (B) Tumors were extracted from mice and representative tumors photographed. A scale bar up to 2 cm is shown for reference. (C) Tumors were measured by a caliper daily over the course of the experiment. (D) Tumors size and (E) tumor mass were measured at the

time of sacrifice. Black bars represent the mean of measurements. (F) Mice weights were recorded over the course of the experiments. The colored line represents the relative weight while the colored bars represent the standard error for weight. Values were given as mean ± SEM. * $P < 0.05$

Supplemental Fig 3.

CDDO-2P-Im affect multiple pathways to reduce proliferation and induce apoptosis of RPMI-8226 myeloma cells. (A) A volcano plot of differentially expressed genes between 0.4 μM CDDO-2P-Im and control is shown. (B and C) GSEA analysis was performed for 0.1 μM CDDO-2P-Im and control. The top upregulated and downregulated pathways by Family-wise error rate (FWER) value are shown. (D and E) GSEA analysis was performed for 0.4 μM CDDO-2P-Im and control. The top upregulated and downregulated pathways by Family-wise error rate (FWER) value are shown. (F) UPR gene expression changes from RNA-Sequencing of RPMI-8226 shows activation of the UPR only at higher concentrations of CDDO-2P-Im. Values were given as mean ± SD. All qRT-PCR values were normalized to GAPDH, * $P < 0.05$ and ** $P < 0.01$, compared with control.

Supplemental Fig. 4.

High CDDO-2P-Im treatment slightly elevated ROS levels. (A) RPMI-8226 cells were incubated with control or treatment for 6 hours. Reactive oxygen species (ROS) was measured with CellROX™ Green Reagent and analyzed with flow cytometry. Representative panels of ROS and forward scatter (FSC) were shown. (B) Average of percent ROS positive cells was shown for each treatment. 0.4 μM CDDO-2P-Im showed

elevated ROS positive cells but the vast majority cells are ROS negative. Values were given as mean ± SD. * $P < 0.05$ and ** $P < 0.01$, compared with control.

Supplemental Fig. 5.

DARTS assay confirms LonP1 is a binding target of CDDO-2P-Im. DARTS was used to assess LonP1 can be detected as a binding target of CDDO-2P-Im. RPMI-8226 cells were lysed and incubated with DMSO or 50 μM of CDDO-2P-Im for 30 minutes. Then lysates were treated with buffer or increasing concentrations of pronase up to 0.025 mg/ml. Lysates were incubated for 15 minutes with pronase on ice and then Protease inhibitor was added to stop the reaction. Western blot was performed to investigate cleavage of LonP1 and β-actin. CDDO-2P-Im was able to protect LonP1 in the high pronase treatment.

Supplemental Fig. 6.

CDDO-2P-Im and tunicamycin activate UPR in myeloma cells. ARH-77 cells were treated with 0.4 μM CDDO-2P-Im or 2.5 μM tunicamycin for 6 hours. Cells were extracted for RNA and prepared for qRT-PCR. Values were given as mean ± SD. All qRT-PCR values were normalized to GAPDH, * $P < 0.05$ and ** $P < 0.01$, compared with control.

Supplemental Fig. 7.

ISRIB treatment does not affect cell viability. RPMI-8226 cells were treated with control or various concentrations of ISRIB for 24 hours. Cells were then evaluated for cell viability by CellTiter-Glo. Values were given as mean ± SD.
